# David Hubel (1926–2013): the man who developed our understanding of vision

**DOI:** 10.1007/s10072-014-1638-3

**Published:** 2014-01-31

**Authors:** Andrzej Grzybowski, Krzysztof Pietrzak

**Affiliations:** 1 Department of Ophthalmology, Poznań City Hospital, Poznan, Poland; 2 Department of Ophthalmology, University of Warmia and Mazury, Olsztyn, Poland; 3 Department of Orthopaedics and Traumatology, University of Medical Sciences, Poznan, Poland

**Keywords:** Vision, Visual cortex, Neurophysiology

**Fig. 1:**
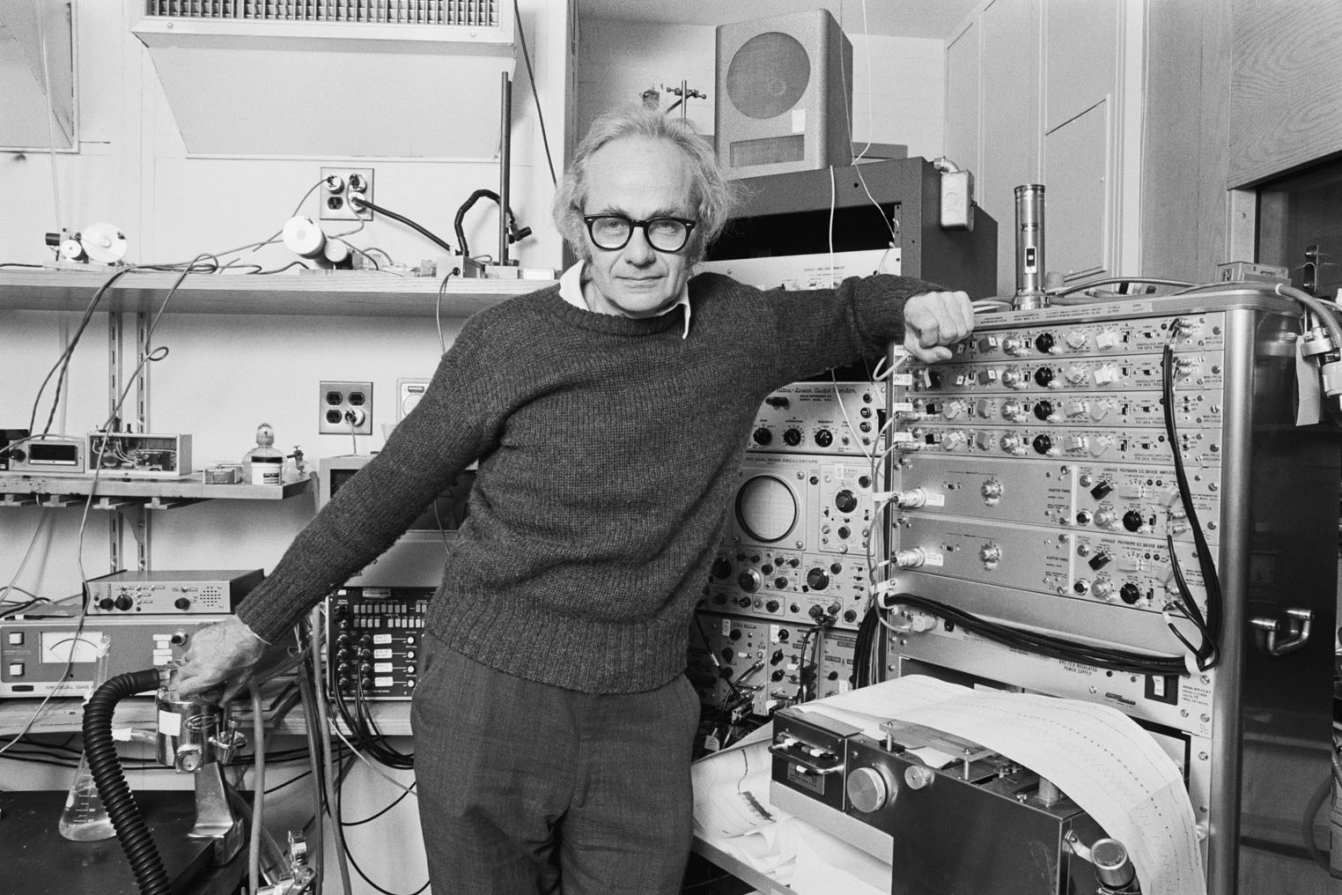
David Hubel (1926–2013)

David Hubel (Fig. 1) was born on the 27th of February 1926 in Windsor, Ontario, Canada. His parents were American emigrants; his father was a chemist. In 1929, they moved to Montreal. From his early age, Hubel, following his father’s footsteps, showed interest in science, especially chemistry and electric engineering. After graduating from Strathcona Academy located on the Montreal suburbs, he began his studies at the McGill University in Montreal. Initially he studied physics and mathematics than medicine [1].

After graduation and apprenticeship, he took 2 years residency in neurology. Already in Canada, under the direction of Herbert Jasper, he developed his interests in neurology. In 1953, he received American, while simultaneously retaining Canadian citizenship. In 1954 he moved to America, where in Baltimore he began residency in neurology at the John Hopkins University School of Medicine.

In 1955, he was drafted into the military service, where fortunately he could devote himself to scientific research. He started working at the Walter Reed Army Medical Center in Washington. Both, his specialization and the long-time interest in electronics drove him to conduct research on the nervous system. Initially he engaged in research on the cord, than the cerebral cortex. Together with a team of neurophysiologists he conducted studies on the visual cortex in sleeping and awakened cats. Hubel invented the modern tungsten microelectrode, supported by a hydraulic control unit that allows detection of signals from that area. Hubel noticed that various light signals stimulate individual neurons whereas others are left inactivated. In 1958, Hubel returned to John Hopkins where he began working with a team under the leadership of Stephen Kuffer (1913–1980). There he had begun a close cooperation with Torsten Wiesel, with whom he discovered selectivity and columnar organization in striate cortex. The research was conducted again placing a microelectrode in the striate cortex of cats. The animals were subjected to subsequent images, light, and dark. Individual neurons responded differently to diverse light intensity and graphic elements such as rectangles, with various angles of inclination. Some fired rapidly with lines at one angle while others were activated with light at a different one. Different images caused the neurons activity to change. These findings led Hubel and Wiesel to hierarchize striate cortex cells [2, 3].

In 1959, a publication authored by Hubel and Wiesel appeared on this subject [4]. It showed the results of research conducted on 24 lightly anesthetized cats whose eyes were opened using wire clips. The authors provided cats with light impulses to one or both eyes. The signal was picked up in single cells of the striate cortex. The cells of the striate cortex were activated in continuous light and the deepening of anesthesia decreased the degree of cell activity. The area of the retina, which caused the activity of a single field in the striate cortex, was later called the receptive field. Within these fields Hubel and Wiesel distinguished excitatory and inhibitory regions. A single light stimulus, illuminating the receptive field of the retina, as a source of scattered light was relatively ineffective in stimulating the entire striate cortex due to the antagonistic effect of excitatory and inhibitory regions.

Effective cortical stimulation depends on the size and shape of light stimuli—in accordance with the distribution of excitatory and inhibitory regions within the receptive area of the retina. Hubel and Wiesel also noted that the intensity of cortical response also depended on the direction of the light source movement. They examined 45 individuals within the cortex—corresponding to the respective regions in the retina—36 were driven from only one eye, 15 from the ipsilateral eye, 21 from the contralateral and the remaining 9 from the two eyes independently. Thy eyes of some cats were equally sensitive; in others the dominance of one eye over the other was noted. The fields within the striate cortex, which were sensitive to the stimuli from both eyes, corresponded to the receptive fields with a very similar construction in both eyes. Also their location in the retina was coherent as to the shape, size and the direction of the movement of light [5].

Neurons which received signals that was strictly determined as to the shape and direction of movement were called by Hubel and Wiesel, simple cells; those which were more versatile, complex cells.

This way the scientists, step by step, made the discovery that the striate cortex is organized in several columns, specialized in processing light signals. As a result the visual cortex became the first mapped area of the brain.

In 1959, Kuffer along with the entire team composed of nine families including that of Hubel, moved to Harvard University in Cambridge. The group closely collaborated at Harvard, and after 5 years created a separate Department of Neurobiology where they continued research and once again undertook the notion of vision. After suturing an eye of a kitten it turned out that the eye turned blind as a result of lack of striate cortex stimulation. On the other hand they demonstrated the flexibility of the cortex where the temporary closure of one eye permanently shifts the ocular dominance of neurons in the striate cortex to the eye that remains open. This led them to the conclusion on the importance of light impulses on the development of visual cortex. This also led to the discovery of the ocular dominance columns within the visual cortex. In addition, the dominance of one eye over the other proved normal. They also discovered that covering one eye causes lack of proper development of the areas responsible for binocular vision. These studies have altered the way ophthalmologist perceive the most beneficial time for surgical treatment of eye diseases such as congenial cataract and strabismus. Prior to Hubel and Wiesel’s discovery, the decision on the surgical treatment of these conditions was postponed; contemporarily surgical treatment is done in early childhood [5].

Since 1965, Hubel worked as a Professor of Physiology and since 1968 as a Professor in Neurobiology at the Harvard University. In 1979 he was awarded the Dickson Prize. In 1981, together with T. Wiesel, he was awarded the NN “for their discoveries concerning information processing in the visual system”. At Harvard, Hubel held lectures until his retirement in January 2013. He was also the laureate of 12 honorary doctorates.

David Hubel was married to Shirley Hubel and they were an extremely harmonious marriage. They had three sons. Hubel was a man of many interests, he knew several languages, he was interested in music, he played the piano and the flute. He enjoyed pottering about how he conducted astronomical observations, played tennis and squash and liked skiing.

David Hubel was an extremely liked and sociable person who was characterized by great sense of humor.

David Hubel died of kidney failure on September 22, 2013 in Lincoln, MA.
